# News Items of General Interest

**Published:** 1914-07

**Authors:** 


### News Items of General Interest.

Senior Surgeon Joseph H. White, U. S. P. H. S., arrived in Panama, May 1, to begin the study of hookworm in Central America.

Surgeon General William C. Gorgas, U. S. A., received the honorary degree of D. Sc. from Princeton University June 16th, ult. On the following day Yale University conferred upon him the honorary degree of Doctor of Laws.

It has been arranged by the General Education Board to pay in a lump sum the recent grant of $1,500,000 to the Johns Hopkins Medical School for the establishment of the William H. Welch Endowment for Clinical Education and Research.

A campaign against mosquitoes is to be carried on in Atlantic City, for which $26,000 is available. Though the swamps of Cape May county practically preclude the possibility of entirely ridding Atlantic City of mosquitoes, conditions should certainly be much improved.

Oregonians are beginning to appreciate the worth of the doctor in politics in nominating Dr. C. J. Smith, of Portland, as Governor of their State. Dr. Smith's popularity has not been confined to politics alone, as he is likewise prominent in his profession, being an ex-President of the Oregon State Medical Association.

---

THE Public Health Service, through Secretary of the Treasury McAdoo, has asked Congress to appropriate $100,000 to be used to prevent the entrance of contagious diseases into the United States, especially from Mexico. The regular fund for the purpose is exhausted.

---

THE Wisconsin Supreme Court on June 17th sustained the so-called Wisconsin "eugenics" law, which provides that couples intending to marry must in order to obtain a license present certificates from physicians stating that they are physically fit. The Supreme Court thus reverses the decision of the lower court, which had held that the law was unconstitutional.

---

THE State Board of Charities and Corrections has formulated plans for an investigation of the problem of the feeble-minded in Virginia. The Board was commissioned to investigate the situation and be prepared to report a plan of remedy to the next General Assembly. For this purpose $3000 a year was appropriated for the next two years. The work will be commenced July 1st. It is at present the purpose of the Board to begin the investigation with an examination of the inmates of prisons and reform schools.

---

THE President of the French Republic has conferred upon Dr. Simon Flexner, Director of the Laboratories of the Rockefeller Institute for Medical Research, the Cross of Chevalier of the Legion of Honor, in recognition of his services to medical science, his discoveries and his administration of the Rockefeller Institute. In 1909, during an epidemic of spinal meningitis in France, Dr. Flexner sent a quantity of anti-meningitis serum to the Pasteur Institute in Paris, by reason of which the epidemic was stopped. Special notice is taken of this in the award.

---

EXERCISE FOR THE BABY.—Marianna Wheeler, author of "The Young Mother's Handbook," tells of an incident in her experience when a baby which had every care failed to thrive. Each day's routine—its clothing, its food, its outings—was studied carefully in order to find, if possible, the cause of its frequent colds. It was decided that the conditions surrounding it seemed perfect, with one exception—an almost total lack of exercise. Most of its waking hours were spent out of doors wrapped up under heavy coverings. As soon as a series of leg and arm exercises were started the baby's health began to improve and his chronic colds disappeared.—*Woman's Home Companion.*

---

All the pulpits of Atlantic City and about fifty in Philadelphia were filled by prominent physicians and surgeons, Sunday, June 21st. In commenting editorially on this the *Public Ledger* said: "It is a great service that eminent members of the American Medical Association rendered when fifty of them addressed as many churches. * * * The Medical Association and the Bar Association have each a reputation to conserve. The public looks to them to be defenders of the faith as it has been delivered to the leaders of their professions. Nor is the popular expectation on the whole disappointed. Increasing attention is paid to preventive medicine, by which medical altruism in checking the ravages of disease would diminish its own practice. * * * Widespread intelligence of causes and cures is urged, and for legitimate education and authentic announcements the publicity which newspapers effectually secure."

---

An important conference has taken place between the Local Government Board, the City Corporation and the Port of London Authority on the subject of rat plague and the risk of human infection. That the danger is a serious one is evidenced by the fact that no fewer than seven out of the 4358 rats bacteriologically examined at the instance of Dr. Herbert Williams, medical officer of health for the Port of London, last year, were found to be infected with plague.

It was pointed out at the conference that if more than two cases of human plague were reported London would be declared an "infected port," and the financial loss to the shipping trade would be very large. Three sets of precautions were suggested:

1. That all the warehouses, dwelling houses and offices in the docks in which food was placed or stored should be made ratproof.

2. That all refuse and waste matter which formed harborage for rats should not be disposed of round the warehouses.

3. That the present type of sanitary conveniences be replaced by others inaccessible to rats.

---

Considerable interest is being taken in a report on the curative value in certain common conditions of raw potato juice, which recently appeared in the *Lancet*, and which, since then, has been published in book form. The author has for some time utilized as a remedy potato juice made by squeezing the raw vegetable in a powerful press as the basis for various preparations, notably liniments and plasters. This juice, to which the name of "extractum solani liquidum" has been given, is said to contain large quantities of potash salts. It is claimed that the application of the preparations of raw potato have a rapidly curative action in various ailments of the rheumatic kind, including lumbago, mild joint rheumatism and synovitis. For lumbago and rheumatism it is recommended while the treatment may further be aided by the special plasters. For severe bruises and sprains it is advised that the hot poultices referred to above should be first resorted to, when it may be expected that the pain will quickly be relieved, and the swelling go down. In gout it would appear that potato juice applied in the right manner will quickly relieve pain and swelling, although it may not be able to relieve the general constitutional symptoms. In the article referred to seven cases of acute gout are recorded, in which the remedy was applied with success.

---

EDUCATIONAL MEASURES.—The Council on Public Health of the A. M. A. made three very valuable recommendations:

The first calls for a thorough investigation of public health conditions in the United States for securing accurate information on all phases of the problem of bettering general conditions. To accomplish this it is contemplated to plan a survey of all public health activities, to assist health officers and to aid in improving general health conditions in cities. This work is now under way.

The second is to use every available means for the education of the public by every possible avenue to an understanding of the enormous advances in scientific medical knowledge during the last generation. The press bureau, speakers' bureau and the exhibition features are to be continued and enlarged to this end. The sum of $30,000 was expended for this purpose last year, and will be materially increased during the coming year.

The third seeks the crystallization of educated public sentiment in securing necessary public health laws, regulations and ordinances, which will render possible a conservation of human life commensurate with advancing knowledge, and which will render such laws effective through the only force available in this country—public opinion. For this purpose the council has established a medico-legal bureau for the special study of public health laws, and to assist legislators in forming the most advantageous statutes for the good of the public.

